# c-Src Binds to the Cancer Drug Ruxolitinib with an Active Conformation

**DOI:** 10.1371/journal.pone.0106225

**Published:** 2014-09-08

**Authors:** Yankun Duan, Lin Chen, Yongheng Chen, Xue-gong Fan

**Affiliations:** 1 Department of Infectious Diseases & Laboratory of Structural Biology, Key Laboratory of Cancer Proteomics of Chinese Ministry of Health, XiangYa Hospital, Central South University, Changsha, Hunan, China; 2 Molecular and Computational Biology Program, Department of Biological Sciences, University of Southern California, Los Angeles, California, United States of America; University of Washington, United States of America

## Abstract

The cancer drug Ruxolitinib is a potent janus kinase inhibitor approved for the treatment of the myeloproliferative neoplasms. In addition, Ruxolitinib has weak inhibitory activity against a panel of other kinases, including Src kinase. There is no structural information of Ruxolitinib binding to any kinase. In this paper, we determined the crystal structure of c-Src kinase domain in complex of Ruxolitinib at a resolution of 2.26 Å. C-Src kinase domain adopts the DFG-in active conformation upon Ruxolitinib binding, indicating Ruxolitinib is a type I inhibitor for c-Src. Ruxolitinib forms two hydrogen bonds with Met341, a water-mediated hydrogen bond with Thr338, and a number of van der Waals contacts with c-Src. Ruxolitinib was then docked into the ligand-binding pocket of a previously solved JAK1 structure. From the docking result, Ruxolitinib also binds JAK1 as a type I inhibitor, with more interactions and a higher shape complementarity with the ligand-binding pocket of JAK1 compared to that of c-Src. Since Ruxolitinib is a relatively small inhibitor and there is sizeable cavity between Ruxolitinib and c-Src ligand-binding pocket, we propose to modify Ruxolitinib to develop more potent inhibitors to c-Src.

## Introduction

Protein kinases catalyze the transfer of a phosphoryl group from adenosine triphosphate (ATP) to serine, threonine or tyrosine residues of its substrate proteins[1]. Such posttranslational modifications serve as a mechanism to modulate enzymatic activity or molecular interactions of substrate proteins in response to endogenous and exogenous signals[1]. Phosphorylation plays a critical role in signal transduction and regulates numerous cellular processes including cell adhesion, invasion, proliferation, survival and angiogenesis[2]. Over-expression or mutations of protein kinases can lead to a variety of human diseases such as cancer and autoimmunity. Protein kinases are therapeutic targets for the treatment of human diseases[3]. A prototypical example, Imatinib, targets BCR-Abl, a constitutively active form of the Abl kinase that leads to chronic myeloid leukemia (CML), and is very successful in the treatment of this disease[4].

Because of a high degree of sequence conservation within the kinase domain, it is not surprising that most kinase inhibitors tend to have limited target specificity. Off-target effects can be beneficial in some cases, but can lead to side effects in other cases. Every kinase inhibitor has its unique and highly unpredictable target spectrum [5]. Understanding the mechanism behind the target specificity is an important goal that would enhance the use of existing kinase inhibitors and benefit the process of inhibitor development. For example, the structural information of Imatinib binding kinase was not only studied in complex with its intended target kinase Abl[6], but also studied in complex with other kinases, including c-Src, Lck, p38[7]–[9]. These studies greatly help us understand the basis of kinase inhibition, selectivity, and potential off-target effects. In addition, these studies provide a structural scaffold for the development of new kinase inhibitors of different kinases.

Protein kinase inhibitors are typically divided into three subtypes: type I, type II and type III inhibitors. Type I inhibitors occupy the pocket primarily filled by ATP, and a catalytically important Asp-Phe-Gly (DFG) motif is kept in active conformation (referred to as DFG-in conformation). A typical example of a type I kinase inhibitor is the second-generation BCR-Abl inhibitor, Dasatinib. Type II inhibitors, such as Imatinib, occupy the ATP-binding pocket and an additional region, and the DFG motif is rotated by ∼180° with respect to the active conformation (referred to as DFG-out conformation)[10]–[12]. Type III inhibitors bind regulatory domains outside the ATP-binding pocket, and modulate the kinase activity in an allosteric manner. Because the amino acids outside the ATP-binding pocket are less conserved relative to those in the pocket, it has been proposed that it might be easier to achieve kinase selectivity with type II or type III inhibitors.

A single residue within the ATP-site of protein kinases, termed the gatekeeper, plays an important role in forming the specificity pocket, and controls sensitivity to a variety of small molecule inhibitors. The gatekeeper residue varies among different protein kinases. Some kinases have a small residue (e.g. Thr, Ala, or Gly) at this position, and are readily targeted by structurally diverse classes of inhibitors. Other kinases possess a larger residue (e.g. Phe) at this position, and are more resistant[13]. Mutation of the gatekeeper residue is a common mechanism of resistance to kinase inhibitors. For example, substitution of BCR-Abl gatekeeper Thr-315 to Ile has led to resistance to Imatinib[14]–[16].

Ruxolitinib is a potent janus kinase inhibitor for the treatment of the myeloproliferative neoplasms (MPNs)[17]. It has potent inhibitory activity against JAK1 (IC_50_ = 3.3 nM) and JAK2 (IC_50_ = 2.8 nM), moderate activity against TYK2 (IC_50_ = 19 nM) and weak activity against JAK3 (IC_50_ = 428 nM) and a panel of other kinases, including Src kinase [18], [19]. JAK2 mutations and activation play a fundamental role in the etiology of human MPNs. For example, about half of patients with MPNs carry a gain-of-function mutation in the JAK2 gene (JAK2 V617F)[17], [18]. Ruxolitinib inhibits the dysregulated JAK2 signaling pathway, and showed significant benefits in clinical trials. In 2011, Ruxolitinib was approved for the treatment of intermediate or high-risk myelofibrosis[20].

There is no structural information of Ruxolitinib binding to any kinase. In this paper, we investigate the structural basis for Ruxolitinib binding to a kinase, using c-Src kinase domain as a prototypical system. By determining the structure of a Ruxolitinib complex at 2.26 Å resolution, we showed that the c-Src kinase domain adopts the DFG-in active conformation. Ruxolitinib forms two hydrogen bonds with Met341, a water-mediated hydrogen bond with Thr338, and a number of van der Waals contacts with c-Src. We then docked Ruxolitinib into the ligand-binding pocket of a previously solved JAK1 structure. From our docking result, Ruxolitinib also binds JAK1 as a type I inhibitor, with more interactions and higher shape complementarity with the ligand-binding pocket of JAK1 than that of c-Src. Since there is sizeable cavity between Ruxolitinib and the c-Src ligand-binding pocket, it is highly possible to modify Ruxolitinib to develop more potent inhibitors to c-Src.

## Methods

### Protein Expression and Purification

Chicken c-Src (residues 251-533) was prepared as previously described [21]. In brief, the protein was expressed with a 6× His-tagged in Escherichia coli BL21DE3 cells, in the presence of the YopH phosphatase. The protein was first purified by Ni-NTA beads. The 6× His-tag was then removed by TEV cleavage. After cleavage, anion exchange chromatography and size-exclusion chromatography were utilized to further purify the protein. Protein in 50 mM Tris(pH 8.0), 100 mM NaCl, 5% Glycerol, 1 mM DTT was concentrated to 10 mg/ml and flash frozen for storage at −80°C.

### Crystallization

The c-Src/ruxolitinib crystals were obtained at 18°C using the hanging drop vapor diffusion method. The c-Src/ruxolitinib complex was mixed in a solution of 170 µM c-SRC, 2000 µM ruxolitinib, 10% DMSO, 50 mM Tris (pH 8.0), 100 mM NaCl, 5% glycerol, 1 mM DTT. The reservoir solution contained 0.1 M MES (pH 6.4), 2% glycerol, 8% PEG 4000, 50 mM sodium acetate, 10 mM MgCl2. Crystals were cryoprotected in reservoir solution plus 20% glycerol and 2000 µM ruxolitinib. Crystal belongs to the space group P1 with cell dimensions of *a* = 42.107 Å, *b* = 63.228 Å, *c* = 73.989 Å, α = 79.27°, β = 89.27°, γ = 90.29°.

### Data Collection and Structure Determination

Data was collected at the Shanghai Synchrotron Radiation Facility (SSRF), beamline BL17U, and the Advanced Light Source (Lawrence Berkeley National Laboratory) beamlines 5.0.2 and 8.2.1. Data was reduced using HKL2000[22]. Structure determination was carried out as described previously [23], [24]. Initial phase determination was performed by molecular replacement with Phaser from the CCP4 package[25], using chain A of a previously solved c-Src structure(PDB code 2SRC)[26] as the search model. The structure was refined using Phenix.refine and Coot from the Phenix package[27]. The statistics of the crystallographic analysis are presented in Table 1. Graphical representations of structure were prepared using PyMol (DeLano Scientific, San Francisco, CA). The coordinates and structural factors have been deposited in the Protein Data Bank with accession code 4U5J.

**Table 1:** Data collection and refinement statistics.

	c-Src/Ruxolitinib complex (pdb: 4U5J)
**Data collection**	
Space group	P1
Cell dimensions	
*a*, *b*, *c* (Å)	42.107, 63.228, 73.989
α, β, γ (°)	79.27, 89.27, 90.29
Resolution (Å)	50–2.26 (2.30–2.26) *
*I*/σ*I*	15.9 (2.9)
Completeness (%)	97.3 (90.3)
Redundancy	3.5 (2.4)
**Refinement**	
Resolution (Å)	43.40–2.26
No. reflections	34000
*R*_work_/*R*_free_	0.197/0.236
No. atoms	4516
Protein	4290
Ligand/ion	46
Water	180
R.m.s. deviations	
Bond lengths (Å)	0.009
Bond angles (°)	1.273

*Values in parentheses are for highest-resolution shell.

### Molecular Docking and structural analysis

Molecular docking was carried out using Molsoft ICM-Pro[28]. Ruxolitinib was docked into the ligand-binding pocket of JAK1 (pdb code: 3EYG)[29]. Volume of the ligand-binding pocket was calculated using POCASA [30]. Diagrams of protein-ligand interactions was generated using LIGPLOT[31].

### Kinase Assays

c-Src (diluted in 50 mM Tris pH 7.5, 0.1 mM EGTA, 0.1% β-mercaptoethanol, 1 mg/ml BSA) was assayed against KVEKIGEGTYGVVYK in a final volume of 25.5 µl containing 50 mM Tris pH 7.5, 0.1 mM EGTA, 0.3 mM KVEKIGEGTYGVVYK, 10 mM magnesium acetate and 0.05 mM [33P-γ-ATP] (50–1000 cpm/pmole) and incubated for 30 min at room temperature. Assays were stopped by addition of 5 µl of 0.5 M (3%) orthophosphoric acid, harvested onto P81 Unifilter plates with a wash buffer of 50 mM orthophosphoric acid, and dried in air. The dry Unifilter plates were then sealed on the addition of MicroScint O and were counted in Packard Topcount NXT scintillation counters.

## Results and Discussion

### Ruxolitinib binding to the ATP-binding pocket of c-Src

**Figure 1:**
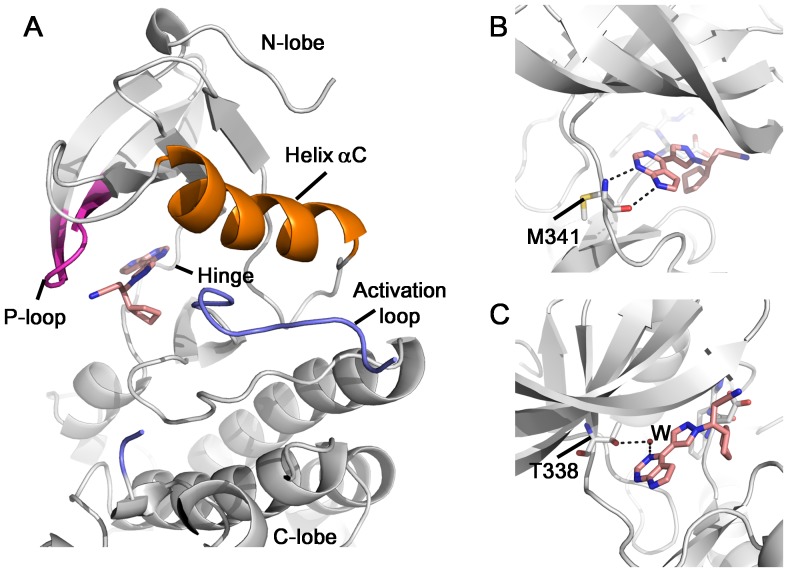
Structure of c-Src in complex with Ruxolitinib. (A) Overall structure of c-Src/Ruxolitinib complex. (B) The pyrrolopyrimidine rings form two hydrogen bonds with the main chain atoms of Met341. (C) Ruxolitinib forms a water-mediated interaction with Src gatekeeper residue Thr338.

To see how cancer drug Ruxolitinib interacts with a kinase, we determined the X-ray structure of c-Src in complex with Ruxolitinib to a resolution of 2.26 Å. The statistics of data collection and model refinement are listed in Table 1. Each asymmetric unit contains two molecules of c-Src. There is well-defined electron density for Ruxolitinib in both c-Src molecules, which appears to be bound with full occupancy (Figure S1). The structure of the c-Src is composed of a bi-lobed architecture (N-lobe and C-lobe) that is typical for protein tyrosine kinases. The ATP binding site of c-Src kinase is located at the folding cleft between the N- and C-terminal lobes, surrounded by the Hinge region, P-loop, Helix aC, and activation loop (Figure 1A). Part of the activation loop (amino acids 412–423) exhibits no electron density, indicating this region is flexible. Therefore this region is not included in the pdb.

Ruxolitinib binds in the ATP-binding cavity of c-Src, with unambiguous electron density observed for the inhibitor (Figure S1). It is orientated such that the pyrrolopyrimidine rings point toward the hinge region, the cyclopentane ring points toward the C-lobe, while the propanenitrile group points toward the P-loop (Figure 1A). The pyrrolopyrimidine rings form two hydrogen bonds with the main chain atoms of Met341 (Figure 1B), as well as a water-mediated interaction with Src gatekeeper residue Thr338 (Figure 1C). Besides the hydrogen bonds, Ruxolitinib also forms a number of van der Waals contacts with c-Src (Figure S2).

**Figure 2:**
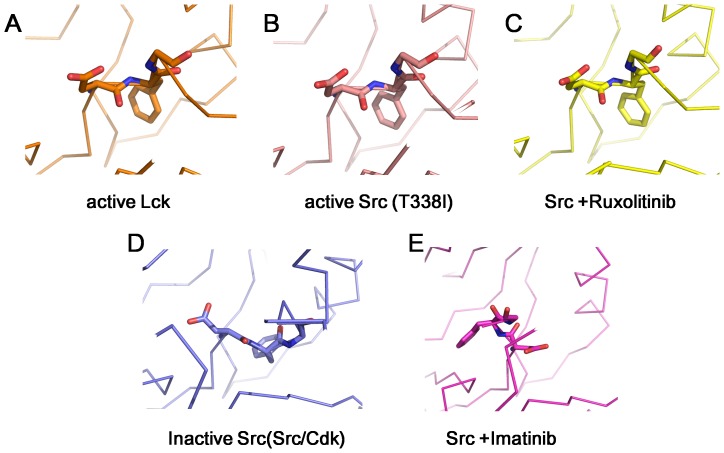
The DFG Motif Conformation. (A) active Lck DFG-in conformation (pdb: 3LCK); (B) active c-Src T338I DFG-in conformation (pdb: 3DQW); (C) active c-Src DFG-in conformation in Src/Ruxolitinib complex (this work); (D) inactive c-Src in the Src/CDK conformation, DFG-intermediate (pdb: 2SRC); (E) inactive c-Src DFG-out conformation in Src/Imatinib complex (pdb: 2OIQ).

DFG motif locates at the N-terminal end of the activation loop, and its conformation plays a pivotal role in kinase activity[32]. DFG-in and DFG-out conformations represent two extreme states of a continuum of possibilities[32]. Intermediate states are observed in some cases[26]. Three distinct conformations of the kinase domains of Src family kinases: a DFG-in active conformation, a DFG-intermediate inactive conformation, and a DFG-out inactive conformation[32]. In the DFG-in conformation (Figure 2A and 2B, pdbs 3LCK and 3DQW), the Aspartate side chain faces into the ATP binding site, and the phenylalanine side chain faces into the protein. In the DFG-out conformation (Figure 2E, pdb 2OIQ), the backbone of the DFG motif is flipped, the aspartate sdie chain faces away from the ATP-binding site, while the phenylalanine side chain occupies the ATP-binding site. For example, the kinase domain of c-Src adopts DFG-out conformation upon binding to Imatinib[33]. In the DFG-intermediate conformation (Figure 2D, pdb 2SRC), the DFG-motif adopts a conformation between the above two conformations. We compared the DFG-motif conformation in Src/Ruxolitinib complex with these three conformations. Apparently, upon Ruxolitinib binding, c-Src kinase domain is kept in DFG-in conformation (Figure 2C).

**Figure 3:**
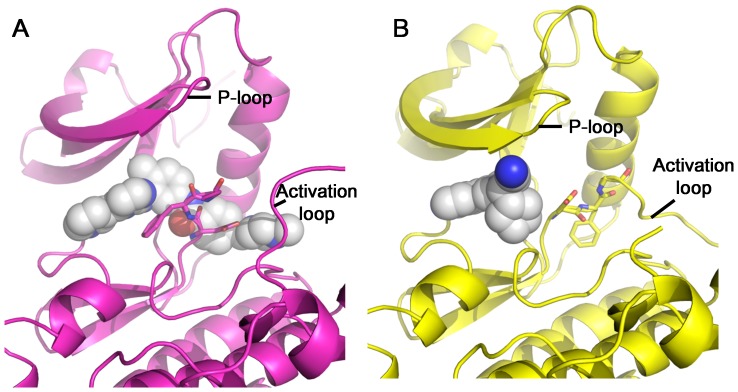
Comparison of pocket occupancy between c-Src/Imatinib and c-Src/Ruxolitinib structures. (A) Pocket occupancy of Imatinib in the c-Src/Imatinib complex (pdb: 2OIQ). C-Src structure is shown in cartoon, colored in magenta. Imatinib is shown in sphere. (B) Pocket occupancy of Ruxolitinib in the c-Src/Ruxolitinib complex (this work). C-Src structure is shown in cartoon, colored in yellow. Ruxolitinib is shown in sphere.

The kinase domain of c-Src adopts different conformations upon binding of different inhibitors. In the c-Src/Imatinib complex, Imatinib occupies not only the ATP-binding site, but also extends beyond the DFG motif and occupies an additional region. The DFG motif is in the DFG-out conformation (Figure 3A). In the c-Src/Ruxolitinib complex, Ruxolitinib only occupies the ATP-binding site, and c-Src adopts a DFG-in conformation. The P-loop moves closer to the C-lobe, leading to a tighter ATP-binding site. This could be due to an induced-fit mechanism (Figure 3B). Based on these observations, Imatinib is a type II inhibitor for c-Src while Ruxolitinib is a type I inhibitor for c-Src.

Most kinase inhibitors are ATP-competitive and belong to type I inhibitors. The ATP-binding pocket is conserved among members of the kinase family. In addition to the ATP-binding pocket, type II inhibitor binds a less-conserved additional pocket. Type II inhibitor acts by inducing a conformation change such that the kinase is no longer able to function. It is proposed that type II inhibitor could achieve better selectivity. Nonetheless, biological efficacy, especially clinical efficacy, will be the ultimate arbiter.

### Comparison of binding pockets in Src and JAK

**Figure 4:**
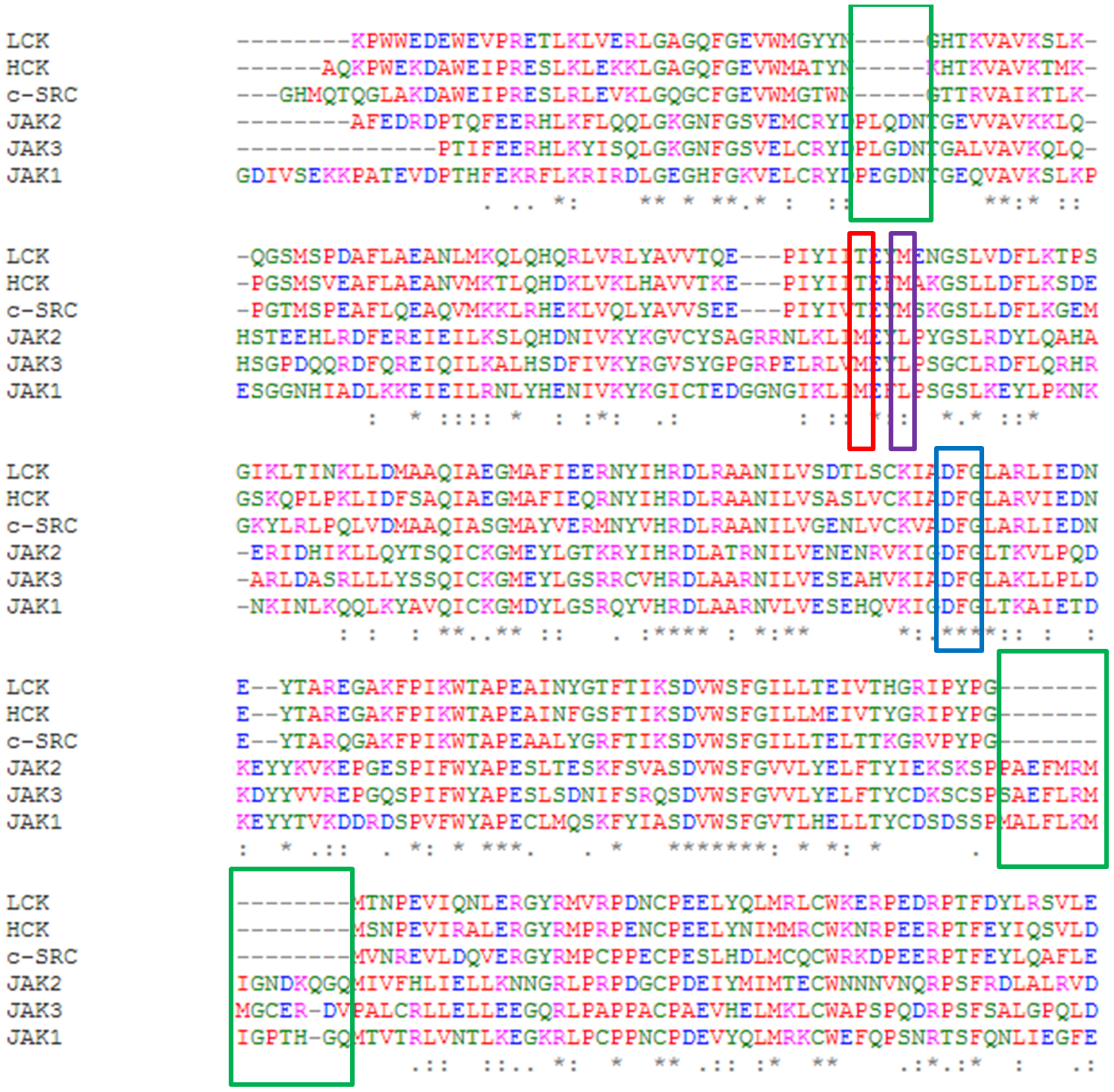
Sequence alignment of LCK, HCK, c-SRC, JAK1, JAK2 and JAK3 kinase domains. The conserved DFG motif is highlighted in blue box; the gatekeeper residue is highlighted in red box; residue corresponding to Met341 in c-Src is highlighted in purple box; several extra stretches of amino acids are highlighted in green box.

Ruxolitinib is a Janus kinase inhibitor, with reported IC_50_ values of 2.8 nM for JAK2, 3.3 nM for JAK1, 19 nM for TYK2, and 428 nM for JAK3[18]. Lck, a Src family kinase, is inhibited by Ruxolitinib with a reported IC_50_ value of 3.6 uM[19]. Using kinase assays, we found that Ruxolitinib inhibited c-Src with an IC_50_ of 2.92 uM (Table S1). Therefore, Src kinases are inhibited by Ruxolitinib to a much lesser extent than JAK kinases. The sequence identity between Src and JAK family kinases is ∼34% in the kinase domain (Figure 4A & 4B). Besides the fact that JAK kinases have several extra stretches of amino acids (Figure 4A, highlighted in green box), We identified several other key differences between Src kinase family and JAK kinase family. Thr338, the gatekeeper residue in c-Src, is replaced with Met in the JAK family; Met 341 of c-Src, the residue forming two hydrogen bonds with Ruxolitinib, is substituted with Leucine in the JAK family (Figure 4A).

**Figure 5:**
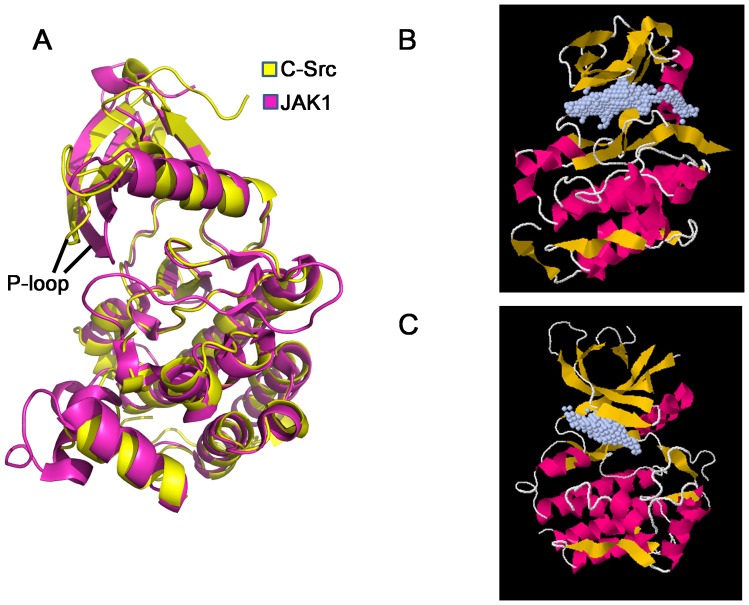
Structure comparison of c-Src and JAK1 structures. (A) c-Src kinase domain (this work) is superimposed with JAK1 kinase domain (pdb: 3EYG) using the Ca atoms of kinase domain as the reference. c-Src is shown yellow, while JAK1 is shown in magenta. (B) Predicted c-Src ligand-binding pocket is shown in grey dots. (C) Predicted JAK1 ligand-binding pocket is shown in grey dots. Ligand-binding pocket is predicted using POCASA[30].

We then superimposed our structure with a JAK1 structure (pdb: 3EYG)[29]. Both kinase domains share a similar overall structure with a RMSD of 2.92 Å. The P-loop of JAK1 is closer to the C-lobe, leading to a tighter pocket, compared to that of C-Src (Figure 5A). We then used POCASA [30] to analyze the ligand-binding pockets of c-Src (our structure) and JAK1 (pdb code: 3EYG). JAK1 has a smaller pocket with a volume of 311 Å^3^, while c-Src has a bigger pocket with a predicted volume of 450 Å^3^ (Figure 5B & 5C).

**Figure 6:**
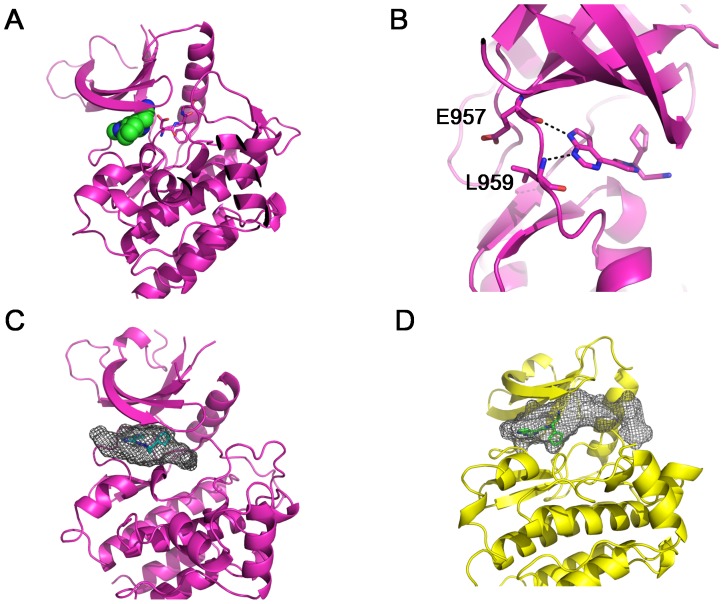
Docking of Ruxolitinib in JAK1 kinase domain. (A) Ruxolitinib was docked into the ligand-binding pocket of JAK1 (pdb: 3EYG). JAK1 kinase domain is shown in cartoon, and colored in magenta. Ruxilitinib is shown in sphere. (B) Predicted hydrogen bonds between Ruxolitinib and JAK1 hinge region. Based on docking result, the pyrrolopyrimidine rings of Ruxolitinib form two hydrogen bonds with JAK1 Glu957 and Leu959. (C) Shape complementarity between Ruxolitinib and JAK1 ligand-binding pocket. (D) Shape complementarity between Ruxolitinib and c-Src ligand-binding pocket. The predicted ligand-binding pocket is shown in grey mesh.

To better understand how Ruxolitinib differentiates these kinase families, we docked Ruxolitinib into a previously solved JAK1 structure (pdb: 3EYG)[29]. The docking result shows that Ruxolitinib only occupies the ATP-binding site, and the DFG motif is kept in DFG-in active conformation (Figure 6A), suggesting that Ruxolitinib is also a type I inhibitor for JAK1. This is consistent with previous finding using competition binding assays[5]. Ruxolitinib is orientated such that the pyrrolopyrimidine rings point toward the hinge region. Instead of forming two hydrogen bonds with Met341 in c-Src, the pyrrolopyrimidine rings form two hydrogen bonds with the main chain atoms of Glu957 and Leu959 (Figure 6B). A major difference between Ruxolitinib binding to c-Src and JAK1 lies on the orientation of the cyclopentane ring. It points toward to the C-lobe in the c-Src/Ruxolitinib complex, while points toward N-lobe when binding to JAK1 (Figure 6B). Another notable difference is that the propanenitrile group of Ruxolitinib makes contacts with JAK1, while it does not interact with c-Src (Figure S3).

There is a high shape complementarity between Ruxolitinib and the JAK1 ATP-binding pocket (Figure 6C), leading to more contacts with the residues surrounding the pocket than that of c-Src (Figure S3). In the c-Src/Ruxolitinib complex, there is a sizeable cavity adjacent to Ruxolitinib in the ligand-binding pocket, especially between Ruxolitinib and the Helix αC of c-Src (Figure 6D). More interactions and higher shape complementarity could be the reason why Ruxolitinib is a more potent inhibitor for JAK kinases than for Src kinases.

Ruxolitiib is a relatively small inhibitor with a molecular weight of 306.37 g/mol. Adding some extra groups to Ruxolitinib could lead to the discovery of new compounds which are more potent inhibitors for c-Src. For example, adding some chemical groups to fill the cavity between Ruxolitinib and Helix αC of c-Src might greatly improve new compound's affinity with Src kinases. However, because of the increase in ligand volume, the new compound would likely to be too big to fill the JAK ligand-binding pocket, therefore could be a poor inhibitor for JAK kinases.

### Conclusions

In this paper, we present a crystal structure of Ruxolitinib bound to the kinase domain of c-Src. Although Ruxolitinib is not a potent inhibitor of c-Src, the crystal structure shows that the c-Src kinase domain is kept in the DFG-in active conformation that is characteristic of type I inhibitor. In addition, we docked Ruxolitinib into the ligand-binding pocket of a previously solved JAK1 structure. From our docking result, Ruxolitinib also binds JAK1 as a type I inhibitor. Compared with its binding to c-Src, Ruxolitinib has more interactions and a higher shape complementarity with JAK1 ligand-binding pocket, which could be the main reason why Ruxolitinib is a much more potent inhibitor against JAK kinases than Src kinases. In our structural analysis, we noticed that there is sizeable cavity between Ruxolitinib and c-Src ligand-binding pocket, suggesting that modifying Ruxolitinib could lead to the development of potent c-Src inhibitors.

## Supporting Information

Figure S1 **Fo-Fc omit map of Ruxolitinib in the c-Src/Ruxolitinib complex.** The electron density is contoured at 2σ and is superimposed with the final model.
(TIFF)

Figure S2 **Schematic diagram of ptotein-ligand interactions in c-Src/Ruxolitinib complex.** Hydrogen bonds are indicated by dashed lines between the atoms involved, while hydrophobic contacts are represented by an arc with spokes. The diagram was generated by LIGPLOT[31] (Supplementary Reference).
(TIFF)

Figure S3 **Schematic diagram of ptotein-ligand interactions in JAK1/Ruxolitinib docking result.** Hydrogen bonds are indicated by dashed lines between the atoms involved, while hydrophobic contacts are represented by an arc with spokes. The diagram was generated by LIGPLOT[31] (Supplementary Reference).
(TIFF)

Table S1 **Kinase assays show that Ruxolitinib inhibited c-Src with an IC50 of 2.93 uM.**
(DOCX)
