# Supplementary figures and images for: c-Src Binds to the Cancer Drug Ruxolitinib with an Active Conformation
Source: PLoS One. 2014 Sep 8;9(9):e106225. doi: 10.1371/journal.pone.0106225 (PMC4157781; doi:10.1371/journal.pone.0106225)

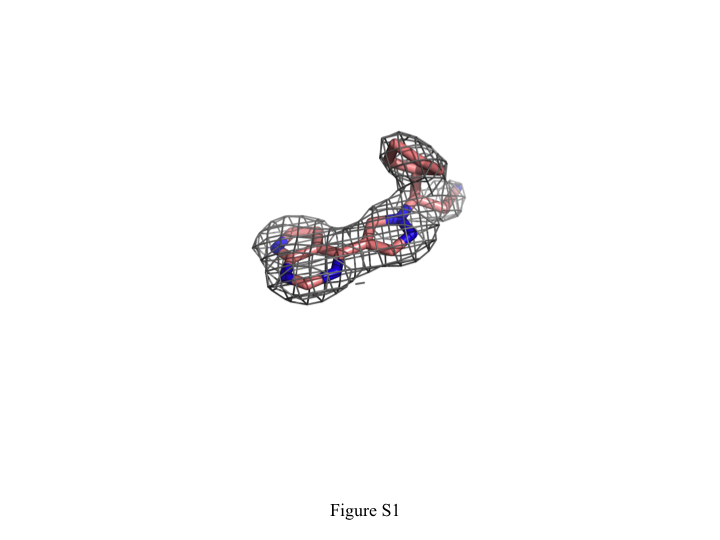

Supplement: Figure S1 — Fo-Fc omit map of Ruxolitinib in the c-Src/Ruxolitinib complex. The electron density is contoured at 2σ and is superimposed with the final model. (TIFF) [file pone.0106225.s001.tiff]

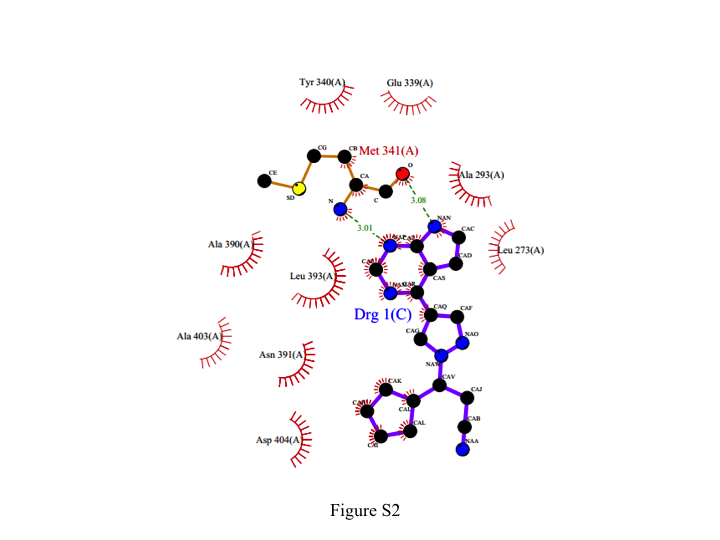

Supplement: Figure S2 — Schematic diagram of ptotein-ligand interactions in c-Src/Ruxolitinib complex. Hydrogen bonds are indicated by dashed lines between the atoms involved, while hydrophobic contacts are represented by an arc with spokes. The diagram was generated by LIGPLOT[31] (Supplementary Reference). (TIFF) [file pone.0106225.s002.tiff]

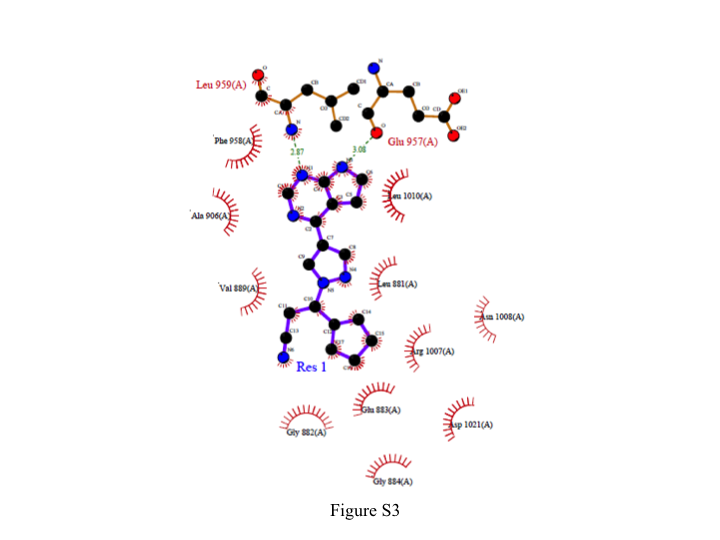

Supplement: Figure S3 — Schematic diagram of ptotein-ligand interactions in JAK1/Ruxolitinib docking result. Hydrogen bonds are indicated by dashed lines between the atoms involved, while hydrophobic contacts are represented by an arc with spokes. The diagram was generated by LIGPLOT[31] (Supplementary Reference). (TIFF) [file pone.0106225.s003.tiff]
