# Supplementary material for: c-Src Binds to the Cancer Drug Ruxolitinib with an Active Conformation
Source: PLoS One. 2014 Sep 8;9(9):e106225. doi: 10.1371/journal.pone.0106225 (PMC4157781; doi:10.1371/journal.pone.0106225)
Supplement: Table S1 — Kinase assays show that Ruxolitinib inhibited c-Src with an IC50 of 2.93 uM. (DOCX) [file pone.0106225.s004.docx]

**Table S1:** Kinase assays show that Ruxolitinib inhibited c-Src with an IC50 of 2.93 uM.

| **Ruxolitinib Conc.(µM)** | **Ruxolitinib Conc.(nM)** | **LOG Conc** | % of Activity | |
| --- | --- | --- | --- | --- |
|  |  |  | 1^st^ Test | 2^nd^ Test |
| **0.003** | 3 | 0.477121 | 101 | 100 |
| **0.01** | 10 | 1.000000 | 87 | 98 |
| **0.03** | 30 | 1.477121 | 84 | 92 |
| **0.1** | 100 | 2.000000 | 93 | 91 |
| **0.3** | 300 | 2.477121 | 66 | 82 |
| **1** | 1000 | 3.000000 | 76 | 69 |
| **3** | 3000 | 3.477121 | 43 | 49 |
| **10** | 10000 | 4.000000 | 29 | 23 |
| **30** | 30000 | 4.477121 | 9 | 13 |
| **100** | 100000 | 5.000000 | 3 | 4 |

* IC50 of Ruxolitinib against c-Src (µM): 3.22 (1^st^ test), 2.61 (2^nd^ test), 2.92 (Average)
